# Progress in Genito-Urinary Surgery

**Published:** 1902-08-09

**Authors:** 


### PROGRESS IN GENITO-URINARY SURGERY.

**Movable Kidney.**—David Wallace[1] gives an account of the discussion on this subject at the last meeting of the Association Française D'Urologie in Paris. All the speakers agreed that the affection is common in women. The average estimate seems to have been that it occurred in 20 per cent. of women. If the left kidney alone suffers there is usually a discoverable cause, such as traumatism, or an intrinsic pathological condition. Guillet considered that fixation of the kidney normally was mainly due to the fibrous capsule. The hollow in which the kidney lies is not so marked in women and is wider inferiorly than in men. The condition is as frequent in nulliparæ as in multiparæ. The corset is not by any means a constant cause as shown, for example, by the fact that Arab women who do not wear corsets frequently suffer from it. Alberran advocates the view that the cause is a hereditary feebleness of the tissues. Guillet explains the greater frequency of mobility on the right side by the absence of the fascia of Toldt on the right, and from the ascending colon dragging upon the right kidney without there being a counteracting force such as the pleuro-colic ligament supplies on the left side. Daret records the results of a post-mortem in a case in which he had sutured the kidney to the posterior abdominal wall by stitches passing through the renal cortex several years previously. He found the kidney firmly adherent by a broad band of fibrous tissue. The discussion strengthens the view that no one cause is sufficient to explain the origin of movable kidney. J. Hutchinson[2] reports two cases of floating kidney in which the chief symptoms were obstructive jaundice and hepatic colic, and supports the view that the chief cause of the symptoms in such cases is downward displacement of the third part or torsion of the second part of the duodenum which in turn affects the common bile duct. In both the cases the jaundice led to the view that the condition was primarily hepatic, and in the first instance laparotomy was performed. J. Scott Riddell[3] contributes a paper on ten cases of movable kidney. The floating kidney, *i.e.*, with a mesonephron, is more common on the left side. In acquired cases in men the writer states it is more commonly on the left side. It is undoubted that menstruation increases the pain, and sometimes the size of movable kidney. With gastric symptoms the affection may closely simulate pyloric obstruction. It seems that Riolan, as long ago as 1649, described the symptoms and complications of movable kidney. It is not more than 20 years since Hahn performed the first nephrorrhaphy. The writer prefers Senn's method of operating, in which, instead of sutures, bridles of gauze are passed over the upper and lower poles of the kidney, and laid over packing running alongside the kidney. The wound heals by granulation. This method, he says, certainly obviates the risk of urinary infiltration, abscess of the kidney, and cirrhosis of the kidney. George M. Edebohls[4] has contributed an exhaustive paper on the technics of nephropexy. He describes how his experience has demonstrated the necessity of removing the vermiform appendix in many patients who need operative fixation of a loose right kidney, and advocates dealing with both organs through the same lumbar incision. The appendix is brought out through the wound by opening the peritoneum to the outer side of the ascending colon, and either inverted entire into the caput coli, after tying off the maso-appendix, or else amputated. It is not a very easy operation, and in 56 cases the author has failed to accomplish it four times. The association of movable kidney with various affections of the bile passages is next considered. The association frequently exists, and many think the kidney is responsible for the trouble in the biliary passages. Direct exploration of the gall bladder, etc., through the lumbar incision, the author maintains is just as satisfactory as exploration through an anterior incision, and he has made the exploration thirty-odd times. The question as to the advisability of dealing with any disease found in the bile passages through the lumbar wound is another matter, and the author says that in some of his cases this would not have been possible. Even if the peritoneum is not opened palpation through it posteriorly will yield valuable information as to the state of gall bladder and ducts. The incision the author uses is the classical one running along the outer margin of the erector spinæ, and should not open the sheath of that muscle. The fibres of the latissimus dorsi are then to be split, not divided, and the sheath of the quadratus opened along the entire extent at the outer margin of the muscle. The difficulty of operating on the kidney is increased in proportion to the length and obliquity of the last rib, and the shortness of the costo-iliac interval. The author has always been able to surmount these difficulties by nicking the outer margin of the quadratus muscle. To avoid post-operative dysæsthesia in the gluteal region the ilio-hypogastric and ilio-inguinal nerves should not be injured. If the nerves have to be divided they should be subsequently sutured. The author removes the renal fatty capsule entirely, also, as in the surgical treatment of chronic nephritis, he strips off the capsule of the kidney, and prefers to fix the organ by passing sutures through the reflected capsule only and not through the renal parenchyma. If sutures are passed through the parenchyma it is very easy to fracture the kidney, and occasionally urinary fistula follows the use of these sutures. The kidney is anchored to the bared surface of the quadratus, and in a position lower than natural. The low position seems to entail no disadvantages. Criticising the tamponade method of exciting adhesions the writer points out the fallacy of the idea that adhesions obtained from granulation tissue are stronger than those from primary union. Most surgeons do not even drain the wounds made. In 68 cases Edebohls has anchored both kidneys at one sitting. Twice he removed the right kidney, and at the same sitting fixed the left one, the patients recovering. The kidney air-cushion is said to be a valuable aid in the operation. The patient is placed prone with the abdomen resting on the air-cushion, which is cylindrical in form, and eight inches in diameter. The ilio-costal space is thus made the most of; both kidneys can be operated upon without any change of position, and delivery of the kidney through the wound for exploration is facilitated. The patients are kept in bed for three weeks after the operation. In the operation now practised by the author, the true capsule of the kidney is stripped off on each side from the external border half-way to the inner border, and four suspension sutures are passed through both the reflected and still attached capsule close to their line of junction. Two of the sutures are placed on the anterior and two on the posterior surface, and each is passed so that a loop is left lying parallel to the long axis of the kidney and immediately under the still attached capsule, but close to the line where it passes into the now unattached part. Thus there are four loops and eight free ends. The kidney is next replaced and each suture end in succession passed through the parieties from within out until they emerge on the surface of the latissimus dorsi, where they are tied after first suturing the muscular and fascial edges of the wound together with a row of buried stitches. Forty-day cat-gut is used for all these buried sutures. The skin is then closed. The mortality of nephropexy is between $1\frac{1}{2}$ and 2 per cent. In estimating results it is to be remembered that absolute fixation is not necessary for therapeutic success. Edebohls considers that the operation has been successful if as a result the kidney cannot be returned by manipulations to its normal position beneath the diaphragm. He has not found a single case among those he has operated upon in which the kidney has become free again, though in several slight apparent mobility was noticeable.

[1] Scottish Med. and Surg. Jour., March, 1902, p. 252. [2] Practitioner, Feb., 1902. [3] Brit. Med. Jour., Feb. 1, 1902, p. 255. [4] Ann. of Surg., Feb., 1902, p. 137.

